# Diagnosis, Management, and Prognosis of Spinal Tuberculosis: A Case Report

**DOI:** 10.7759/cureus.35262

**Published:** 2023-02-21

**Authors:** Arjavon T Talebzadeh, Nojan Talebzadeh

**Affiliations:** 1 Surgery, California Northstate University College of Medicine, Sacramento, USA; 2 Surgery, South County Surgery Center, San Diego, USA

**Keywords:** kyphosis, infection, treatment, tuberculosis, spine

## Abstract

Spinal tuberculosis (TB) is a condition that affects numerous people around the world. The incidence of TB prior to the pandemic was decreasing by about 1.8% per year; however, COVID-19 has complicated this incidence rate leading to an increase of 4.5% in 2020 and 2021. Spinal TB is a rare event in all TB patients. The management could be multifactorial including location, severity, and symptom, and this case demonstrates an example of issues to consider in the diagnosis and management of patients. This is a case of a patient presenting with back pain which was subsequently diagnosed as spinal TB. We will review this patient's presentation and consideration for multifactorial opinions in the literature. This literature review demonstrates that there is no one treatment option available. Providers need to tailor treatment to each individual case. This is an example of a case that illustrates that diagnosis of spinal TB is not straightforward and clinicians may have to make a judgment call and treat prophylactically to prevent a poor prognosis.

## Introduction

Spinal tuberculosis (TB), also known as Pott’s disease, has been around for at least thousands of years. Percival Pott was the first to describe the case of spinal TB in 1779. However, archaeological analysis of mummies suggests that even Egyptian pharaohs were potentially affected by spinal TB [1-3].

Spinal TB is very difficult to treat, and its location makes it a significant risk to the patient’s health. The involvement of the spinal cord can lead to paraplegia or quadriplegia based on the location of the spinal cord. Spinal cord involvement can cause major neurological deficits according to the level affected. High-level infection by* Mycobacterium tuberculosis* can affect the respiratory center in the brain stem leading to significant compromise in the patient's respiratory drive. Spinal TB affects most commonly the thoracolumbar region of the spine [4]. The incidence in this region has been reported to be as high as 56% [5]. It is not unusual to have multifocal involvement [6]. Early infection begins in the metaphysis of the vertebral body before it spreads. In the beginning, the disc space will not be involved. However, the gradual destruction of the affected vertebrae precipitates spinal kyphosis and mechanical instability.

Spinal TB manifests as an extrapulmonary manifestation of tuberculosis - constituting about half of cases of musculoskeletal involvement [6]. Patients will only experience extreme back pain when there is significant bony destruction and deformity. In addition to the constitutional symptoms attributable to *M. tuberculosis* infection, chronic spinal TB presents with severe kyphosis and, in a significant minority of patients, neurologic deficits secondary to mass effect, mechanical instability, and/or stenosis [7]. The kyphosis in chronic spinal TB is on average 15°, but it can be as devastating as 60° in the top 5th percentile of affected patients [8].

It has been shown that early diagnosis is the key to management [9]. Computed tomography (CT) and magnetic resonance imaging (MRI) have been used to discern the extent of lesion and treatment modalities necessary to manage the disease. The differential diagnosis of destructive lesions could range from spinal TB to some metastatic diseases; however, pyogenic and fungal infections could have similar presentations. All these conditions present as destructive spinal lesions with lytic characteristics making it difficult to distinguish radiographically. Clinically these patients have very similar manifestations with patients' presentations ranging from being asymptomatic to pain, fever, and neurologic signs. A positive skin test could be helpful; however, in endemic areas with exposure to TB, this test is less valuable. Biopsy, if possible, with DNA amplification using polymerase chain reaction can better help diagnose this condition. Cultures can also help discern other infections namely fungal or other mycobacterial infections. Fungal cultures use a variety of mediums ranging from solid to liquid. These mediums have a common component namely carbohydrate, nitrogen source, and PH of 5-6. Some examples include Sabouraud dextrose, malt extract, and less commonly brain heart infusion medium. In the case of TB, blood agar is sufficient; however, mediums such as LJ and Middlebrook 7H10 and 7H11 agar have the highest sensitivity and specificities [10,11]. Elevated erythrocyte sedimentation rate (ESR) has been shown to correlate with TB. The reduction of ESR levels can help clinicians assess the treatment progress of the patient [12].

The management of spinal TB has been described extensively. This case however demonstrates the difficulty clinicians face when everything is not straightforward, and suspension of diagnosis may require proactive treatment. The key issue is the location of the lesion (i.e., whether the lesion is located anterior or posterior to the spinal cord) and whether there is neurologic involvement, instability of the spine, and/or presence of kyphosis. Recent recommendations focus on medical management including multiple antituberculosis medications given over a defined period of time.

## Case presentation

A 52-year-old male presented to a local emergency room in Southern California for severe acute-onset back pain. The patient was playing soccer the day before and woke up the following morning with severe lower back pain that was immobilizing. In the emergency room, he reported severe pain and distress. He was completely asymptomatic prior to this event with negative past medical history. He was a middle eastern male with no habits, and he appeared athletic and in good physical shape. He worked as a sales representative for a store and was married for five years. His blood pressure was stable at 128/67 with a heart rate of 95 and was afebrile on clinical evaluation. The pain was localized to the thoracolumbar spine with no signs of inflammation involving nearby skin. There was no neurologic deficit identified on exam of the upper and lower extremities or trunk. The local lumbar region was tender on pressure and movement, but no erythema was noticed on his exam. The patient was placed on intravenous fluids, and morphine was provided for immediate pain control.

**Figure 1:**
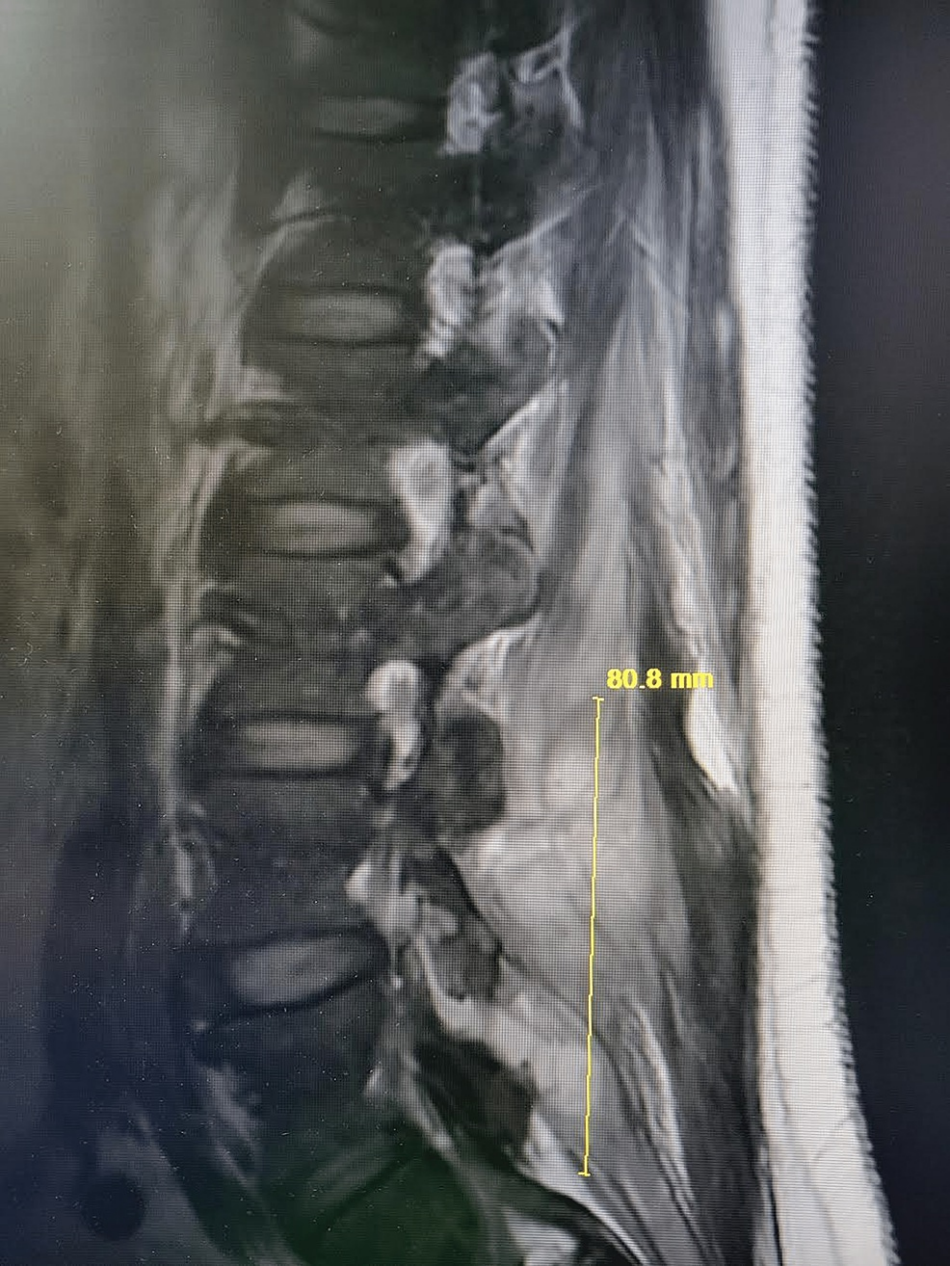
Extensive periarticular enhancement

**Figure 2:**
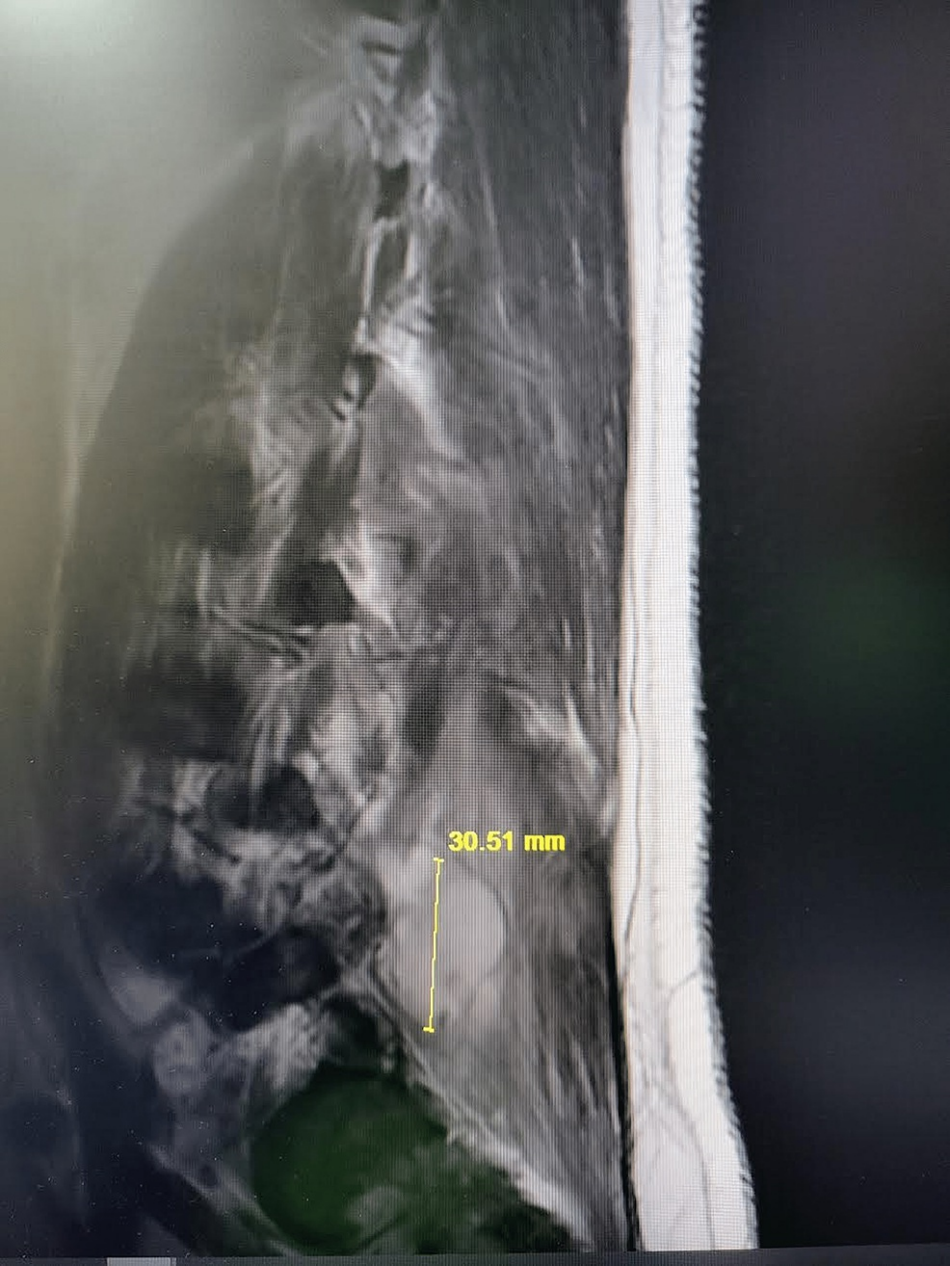
Epidural enhancement

**Figure 3:**
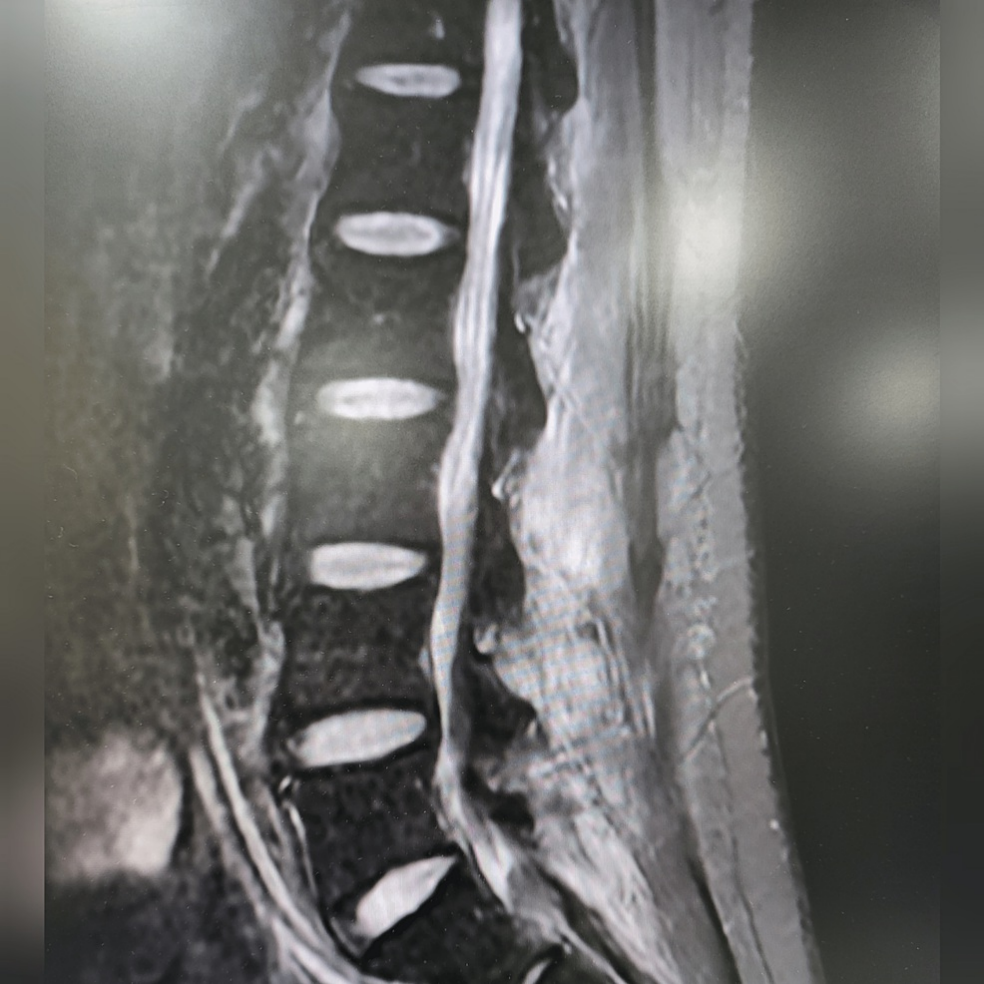
Inflammatory response around spinal cord

Lumbosacral x-ray was significant for small anterior osteophytes at the L5 level. No significant disc space narrowing was noticed. An MRI scan was ordered to better evaluate the questionable soft tissue around the osteophytes. MRI of the spine revealed extensive periarticular enhancement about the right L4-L5 facet of the joint with extension into the epidural space and posterior paraspinal musculature with possible infectious fasciitis (Figure 1). It was also significant for epidural enhancement in thecal sac narrowing, which was moderate at L3 to L5 (Figure 2). Some possible fluid collection was also noted with rim enhancement (Figure 3).

The patient was subsequently taken to operating room for imaging-assisted core biopsy surgery. Biopsy of area close to abnormal region next to L4-L5 was done. Result of core biopsy revealed muscle and fibroadipose tissue with chronic inflammation and no caseation necrosis seen in the sample obtained. Immunostains for AE1/AE3, S100, and desmin were negative. SMA and SATB2 showed patchy positivity. CD138 highlighted some plasma cells. CD3 and CD20 highlighted T and B cells. The patient had a history of BCG vaccination, and therefore positive skin test result was questionable. He was subsequently scheduled for a lung scan to rule out pathologic involvement of the lungs, redo of the core biopsy, and culture for abnormal infections such as TB.

Over the course of the two months patient followed up with clinical spine surgery team and symptoms had improved. His lung CT scan showed negative result; however, due to previous BCG immunization, the positive skin test was expected. The team felt that a second biopsy and culture at different angle and depth may give better result; however, patient was very hesitant to get it done and decision was made to treat patient conservatively with observation and follow up. He was prophylactically placed on Rifampicin, Ethambutol, Isoniazid and Pyrazinamide for two months followed by Isoniazid and Rifampicin for six months. He was stable on his six-month follow up and was placed on continued observation with repeat MRI scan planned in future.

## Discussion

Spinal TB is an uncommon condition seen in less than 1% of TB patients. Being very debilitating, the condition may lead to spinal collapse, precipitating kyphotic deformity. The most involved site is the anterior portion of the spinal column. Destruction of the osseous tissue and abscess formation is the most common finding in spinal TB [3]. It is not unusual for patients to have narrow canals and nerve compression with associated neurologic deficits. These deficiencies could be minor such as paresthesias in specific dermatomes to severe motor innervation deficiencies depending on the area of involvement. Spinal TB can be difficult to diagnose, and biopsy and cultures may not be accessible with standard biopsy techniques. In such cases when the aggressive disease is noticed, Spinal TB and other fungal infections should be considered [13]. Multiple classification systems have been described: Kumar in 1985 [6] based on the stage of disease, Mehta et al. in 2001 [14] based on the surgical technique used for correction, and Oguz et al. based on disc degeneration [15]. Unfortunately, all these classifications are based on limited data and are associated with some deficiencies.

Management of spinal TB is based on medical and surgical treatment. In cases where the lesion is accessible, a surgical approach combined with medical management is the treatment of choice. In cases where the surgical approach is unfeasible or associated with significant possible complications, then medical management using combination antituberculosis medication is considered first [16]. Early diagnosis is important for treatment. The standard combination therapy using Rifampicin, Ethambutol, Isoniazid, and Pyrazinamide for two months followed by Isoniazid and Rifampicin for 6 months is the most recommended treatment [8,10,17,18]. Medical management has been shown to improve the pain symptoms in most patients and has shown that ESR and C-reactive protein will level off over time [19]. The surgical intervention combined with medical management has been shown to be necessary in case of spinal instability or late-onset paraplegia [19,20]. A surgical approach may help prevent late complications in the cases of multi-drug resistant TB or pediatric onset, a time of continued spinal development [21]. Multidrug-resistant TB should be treated with six antituberculosis medications for a minimum of two years [8]. However, these medications do predispose the patient to side effects, warranting close follow-up.

Spinal TB has a favorable prognosis, especially with earlier diagnosis and treatment. There is no definitive recommendation for the treatment of spinal TB. The general consensus seems to be aggressive medical management followed by surgery if the lesion is accessible or if spinal instability exists. In this case, after one attempt at biopsy, the patient was hesitant to get another biopsy due to the location of the lesion. The team decided to proceed with medical management with a combination of Rifampicin, Ethambutol, Isoniazid, and Pyrazinamide for two months and follow up with Isoniazid and Rifampicin for six months. The patient improved on follow-up and was stable and placed on every six months follow-up clinically.

## Conclusions

Spinal TB is a condition that affects a significant number of people around the world. Understanding this disease and management can be crucial for the prognosis of the afflicted patients. The patients can have a variety of presentations and medical, surgical, or combination therapy has to be deployed to treat each patient individually. The general approach is tailored based on the location of the lesion and medical and surgical teams have to work in a coordinated fashion to treat the patients based on clinical findings. It should be mentioned that organized systemic treatment of the population in highly susceptible countries needs to be considered as a preventative method to minimize the spread of this disease. Even though this is a rare condition in modern countries, immigration and travel can predispose patients to this disease, and practitioners should be aware of this condition and treat it appropriately.
